# Effects of Chloroform Extract of *Dryopteris crassirhizoma* on the Ultramicroscopic Structures of *Meloidogyne incognita*

**DOI:** 10.1155/2013/313482

**Published:** 2013-10-24

**Authors:** Ji-quan Liu, Shu-lian Xie, Jia Feng, Jin Cai

**Affiliations:** ^1^School of Life Science, Shanxi University, Taiyuan 030006, China; ^2^Department of Traditional Chinese Medicine, Shanxi College of Traditional Chinese Medicine, Taiyuan 030024, China

## Abstract

In our early experiments, the chloroform extract of *D. crassirhizoma* was demonstrated to contain the highest concentrations of total phloroglucinols among several extract fractions and possessed the most effective nematicidal activity. This study aimed to ascertain the ultrastructural changes in *M. incognita* after treatment with a *D. crassirhizoma* chloroform extract at 1 mg*·*mL^−1^ for 24 h. It was found that the extract exhibited significant destructive effects on the worm's ultrastructure and caused distinctive damage to body surfaces and internal structures. These results will contribute to a deeper understanding of the nematicidal mechanism of *D. crassirhizoma*, as well as in the design of efficient bionematicides.

## 1. Introduction

Root-knot nematodes (*Meloidogyne* spp.) constitute a major group of plant-parasitic nematodes affecting crop production and substantially reducing food quality [1]. *Meloidogyne incognita *(Kofoid and White) Chitwood is one of the most common and important plant-parasitic nematodes in tropical and subtropical regions worldwide [2]. It has been estimated that global losses from this root-knot nematode amount to $78 billion [3]. The management of nematodes is more difficult than that of other pests because nematodes mostly inhabit the soil and usually attack underground parts of plants [4]. Although chemical nematicides are effective, easy to apply, and show rapid effects, they have begun to be withdrawn from the market in some developed countries owing to concerns about public health and environmental safety [5].

Therefore, there is a strong demand to develop more sustainable and environmentally friendly methods for nematode control. One such alternative is seen in the use of plants containing useful secondary plant metabolites. Nematicidal activity has been reported in several hundred plants, including members of the families Asteraceae, Brassicaceae, Leguminosae, Meliaceae, and Liliaceae [6]. Rhizomes from the ferns of *Dryopteris* spp. have been used as antibacterial, vermifuges, and anthelmintics in tapeworm infections [7, 8]. The vermifuge activity of *Dryopteris *spp. has been related to the presence of phloroglucinol derivatives in their extracts [9]. Characteristic phloroglucinol derivatives, such as aspidin and flavaspidic acid, have been reported as major constituents of the genus *Dryopteris*, and they have been found to possess antioxidant, antibacterial, and antitumor promoting activities [7, 8, 10].

In our previous studies, the nematicidal potential of *Dryopteris crassirhizoma* against root-knot nematode *Meloidogyne incognita* was demonstrated in *in vitro* and pot experiments. This nematicidal activity has been related to the presence of phloroglucinol derivatives in petroleum ether, chloroform, ethyl acetate, *n*-butyl alcohol, and water extract fractions from *D. crassirhizoma* on the basis of phloroglucinol analyses and LC_50_ determinations [11]. Compared with other extracts, the chloroform extract from *D. crassirhizoma* contains the highest concentration of total phloroglucinols and displayed the most effective nematicidal activity.

The aim of this present work was to evaluate the nematicidal mechanisms of the chloroform extract of* D. crassirhizoma* against the root-knot nematode *M. incognita* through ultrastructural observations by scanning and transmission electron microscopies.

## 2. Materials and Methods

### 2.1. Plant Material

Rhizomes of *Dryopteris crassirhizoma* were collected from Jiayin County, Yichun City, Heilongjiang Province, China, dried at 50°C, and mechanically pulverized to a particle size of 1 mm. Then, about 10 g of plant material was extracted in 100 mL of 70% aqueous ethanol at 25°C for 3 d and the extracts stirred once every 8 h. After extraction, the solvent was evaporated in a rotary vacuum evaporator, and the residue then dissolved with water (50°C) and sequentially extracted with petroleum ether and chloroform to yield three fractions. The chloroform fraction was evaporated as above, weighed, dissolved in aqueous acetone (5% by vol), and kept as a 2 mg·mL^−1^ stock chloroform extract solution.

### 2.2. Nematode Collection

*Meloidogyne incognita* specimens were collected from naturally infested tomato fields in Qinxu County, Taiyuan City, Shanxi Province, China, and cultured on tomato plants (*Solanum lycopersicum* “*Hengli*”) grown under greenhouse conditions. Egg masses were collected from heavily galled tomato roots and placed in sterile distilled water at 25°C to stimulate juvenile hatching, and the hatched second-stage juveniles were collected. Adult nematodes were collected from the plants by the Baermann method [12].

### 2.3. Nematode Treatment

Collected second-stage juveniles and adult nematodes were incubated at 25°C in chloroform extract solution at 1 mg·mL^−1^ or 5% aqueous acetone as a control [13]. After 24 h, the nematodes were washed four times in fresh distilled water and used for ultrastructural observation by scanning and transmission electron microscopies.

### 2.4. Scanning Electron Microscopy Observation

Nematodes were fixed in 2% glutaraldehyde buffered with 0.1 M sodium cacodylate (pH 7.2) at 4°C for 4 h. Samples were postfixed for 2 h in 1% OsO_4_ in the same buffer and then dehydrated through a graded ethanol series (10%, 20%, 30%, 40%, 50%, 60%, 70%, 80%, 90%, and 100%) at 15 min per step. For scanning electron microscopy, samples were critical point dried with CO_2_, coated with gold-palladium, and observed in a JSM-35 scanning electron microscope at an electron accelerating voltage of 25 kV (JEOL, Ltd., Tokyo, Japan).

### 2.5. Transmission Electron Microscopy Observation

The fixing and postfixing of nematodes were the same as for scanning electron microscopy. Samples for transmission electron microscopy were agar-embedded before dehydration and finally embedded in Araldite 618 resin (Huntsman International, LLC, Salt Lake City, UT, USA). Nematodes were cut with PowerTome XL ultrathin slicing machine (RMC, Boeckeler Instruments, Inc., Tucson, AZ, USA). Thin sections were stained with uranyl acetate, followed by lead citrate, and observed in a JEM-1011 transmission electron microscope at an electron accelerating voltage of 80 kV (JEOL, Ltd.).

## 3. Results

### 3.1. Effects of Chloroform Extract of *Dryopteris crassirhizoma* on Body Surfaces of an Adult Nematode

**Figure 1:**
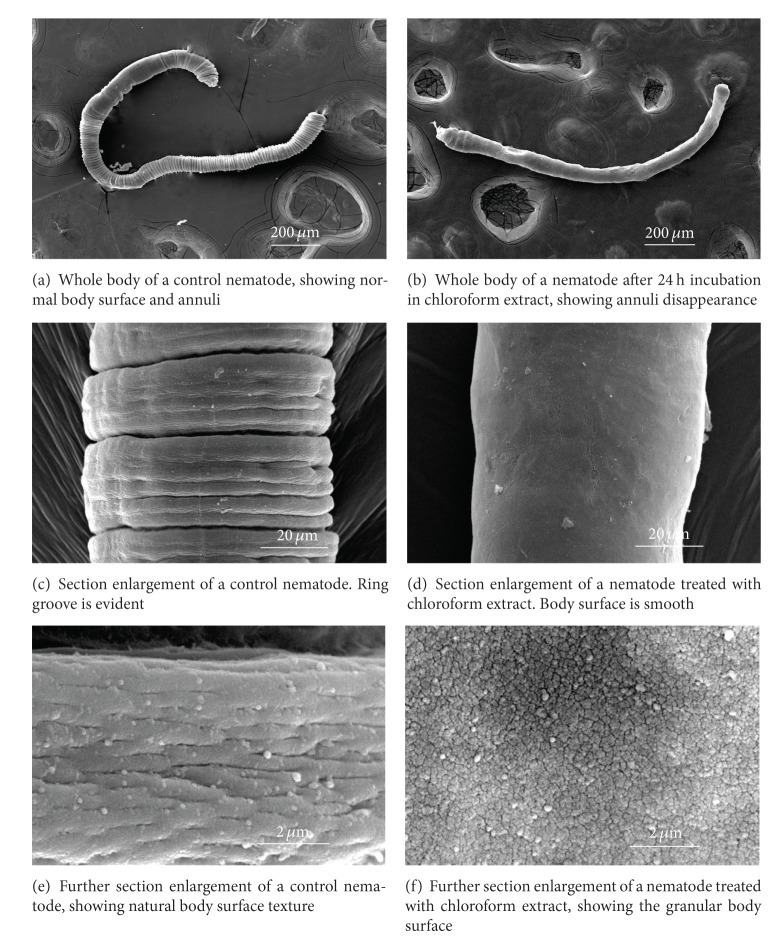
Scanning electron micrographs of *Meloidogyne incognita* (adult).

On a normal or control nematode, the entire body was covered with cuticular tissue and many annuli visible in regular transverse rows (Figure 1(a)). Section enlargements of body surfaces showed the presence of a deeper ring groove occurring every three to five shallow ring grooves (Figure 1(c)) and further enlargement revealed the body surface natural texture to be similar to human skin (Figure 1(e)). In contrast, the body surfaces of a nematode exhibited distinctive and significant damage after treatment with a 24 h *in vitro* exposure to chloroform extract of *D. crassirhizoma* at 1 mg·mL^−1^. After treatment, the typical annuli disappeared, yielding a velvety appearance (Figures 1(b) and 1(d)), and further section enlargement showed that the body surface was composed of many small particles (Figure 1(f)).

### 3.2. Effects of Chloroform Extract of *Dryopteris crassirhizoma* on Body Surfaces of a Second-Stage Juvenile

**Figure 2:**
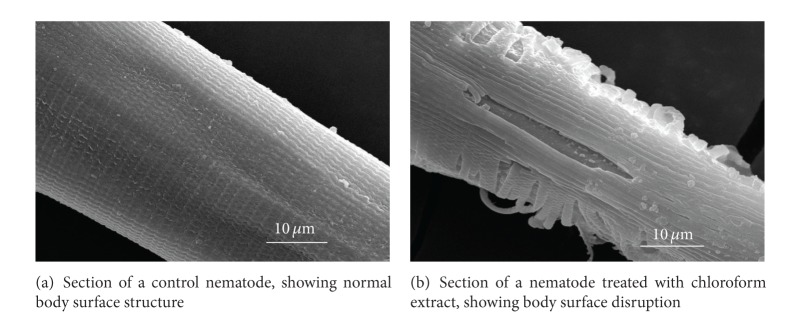
Scanning electron micrographs of *M. incognita* (juvenile).

Figure 2(a) illustrates the observation that the body surfaces of a control juvenile was in good condition and undamaged. In contrast, the appearance of a juvenile treated as described above showed distorted damage, such that the body surface was sloughed off as a flocculent and internal tissue exposed (Figure 2(b)).

### 3.3. Effects of Chloroform Extract of *Dryopteris crassirhizoma* on Cuticle and Hypoderm of an Adult Nematode

**Figure 3:**
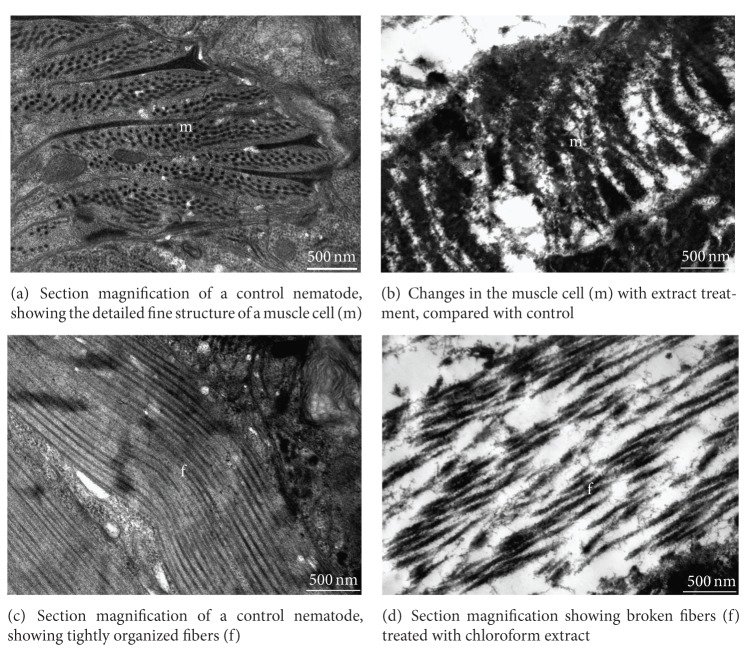
Transmission electron micrographs of cuticle and hypoderm of adult *M. incognita. *

The cuticular architecture comprised two main layers, the external cortical cuticle and basal cuticle layers. The cortical cuticle layer appeared similar to a compact fence structure, which was tightly bounded by the basal cuticle layer (Figure 3(a)). After treatment with chloroform extract, as described above, the normally compact cortical cuticle layer was separated from the basal cuticle layer, broken into flakes, and sloughed off, exposing the basal cuticle layer (Figure 3(b)).

Underlying the basal cuticle was the hypodermal tissue, which, for the most part in a healthy worm, consisted of a single large syncytium, in which the internal architectures were clear, compact, and well organized (Figure 3(c)). After treatment with chloroform extract, as described above, this layer's clear and organized architecture was severely disrupted and appeared blurred, with many gaps formed between the internal tissues (Figure 3(d)).

### 3.4. Effects of Chloroform Extract of *Dryopteris crassirhizoma* on the Muscle Layer and Nerve Fibers of an Adult Nematode

**Figure 4:**
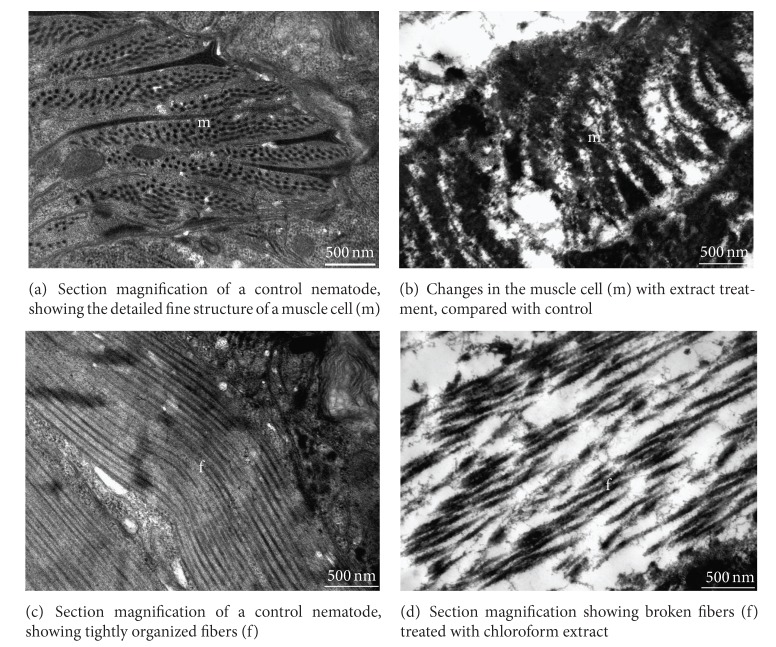
Transmission electron micrographs of muscle and fibers of adult *M. incognita. *

Internal to the hypodermis was the muscular layer, consisting of many large muscular cells, which under healthy conditions showed clear and integrated external contours and clearly visible internal ultrastructures (Figure 4(a)). After treatment with the chloroform extract, as described above, the integrity of muscular cells was compromised, and the structure of the protoplasts severely damaged (Figure 4(b)).

The nerve fibers of a control nematode were not only arranged in an orderly and compact fashion but also appeared very elastic (Figure 4(c)). When treated by chloroform extract, as described above, the nerve fibers were fragmented into many pieces and lacked integrity (Figure 4(d)).

## 4. Discussion

Phloroglucinol derivatives have been reported to have a wide range of biological activities, including antioxidant, antibacterial, and antitumor activities [10]. In addition, it has been reported that phloroglucinol compounds from *Dryopteris* species are used as vermifuges in tapeworm infections [7, 8]. Some phloroglucinol derivatives show schistosomicidal effects *in vitro* against *Schistosoma mansoni* adult worms, producing integumental alterations [14], as well as inhibiting oxidative phosphorylation in rat heart mitochondria and inhibiting the native Mg^2+^-dependent ATPase and ATP–Pi exchange in mitochondria [15, 16]. Phloroglucinol derivatives are typical secondary metabolites widely present in ferns like *Dryopteris*, and those from *D. crassirhizoma* show strong toxicity against *Ascaris lumbricoides*, *Leishmania donovani*, *Taenia solium,* and *T. saginata*, mainly negatively affecting the digestive system and causing central nervous system disorders with tremors and convulsions that can lead to death [17, 18]. In addition, *D. crassirhizoma* is a traditional Chinese medicine commonly used in China and is safe for human and environment when it was applied as a nematicide in nature [17].

The body wall of the nematode *M. incognita* consists of cuticle, hypoderm, and muscle layers. The cuticle lies outermost in the nematode, as a multilayered extracellular structure that completely surrounds the animal, except for small openings into the pharynx, anus, excretory pore, and vulva [12]. The cuticle forms the barrier between the animal and its environment and, in addition to being a protective layer, constitutes an exoskeleton that is important in maintaining and defining the animal's normal shape [19]. For nematodes, the normal cuticle contributes in preventing most harmful chemicals from permeating to internal tissues and producing toxic effects.

In the present study, the chemical component in this solution of *D. crassirhizoma* chloroform extract was found to cause structural alterations of *M. incognita*'s cuticle, separating the cortical cuticle layer from the organism's body surface, which finally gives rise to integumental permeability changes. Harmful chemicals could thus easily permeate into the internal tissues and damage the normal structure and function of hypodermic and muscular tissues.

Nematode movement relies on the somatic musculature that runs longitudinally along the body wall. Neuromuscular synapses occur in the ventral and dorsal cords and employ an excitatory neurotransmitter, acetylcholine, to modulate muscle activity [20]. Acetylcholine is an impulse transmitting substance that bridges the gap of cholinergic synapses. The biological role of acetylcholinesterase in regulating nerve impulse transmissions across cholinergic synapses is by rapid acetylcholine hydrolysis [21, 22]. When this enzyme is inhibited, acetylcholine accumulates at the postsynaptic membrane, leading to continuous stimulation and paralysis of the organism [23]. *Myrtus communis* also contains phloroglucinol-type compounds [24–26] and its chloroform fraction exhibits high inhibition of acetylcholinesterase activity [27].

Here, treatment of a nematode with the chloroform extract resulted in nerve fiber breakage, but it remains unclear that this was caused by extract inhibition of acetylcholinesterase activity, resulting in acetylcholine accumulation and ultimately to continuous stimulation and fiber breakage. More research is required to elucidate the nematicidal mechanisms of this chloroform extract of *D. crassirhizoma *on the root-knot nematode* M. incognita*. 

## 5. Conclusions

The ultrastructural observations of *M. incognita* by scanning and transmission electron microscopies suggested that the chloroform extract of *D. crassirhizoma* had significant destructive effects on the worm's ultrastructure. It indicated that *D. crassirhizoma* might be an efficient bionematicide.
